# Supplementary figures and images for: miR-1/206 downregulates splicing factor Srsf9 to promote C2C12 differentiation
Source: Skelet Muscle. 2019 Dec 2;9:31. doi: 10.1186/s13395-019-0211-4 (PMC6888935; doi:10.1186/s13395-019-0211-4)

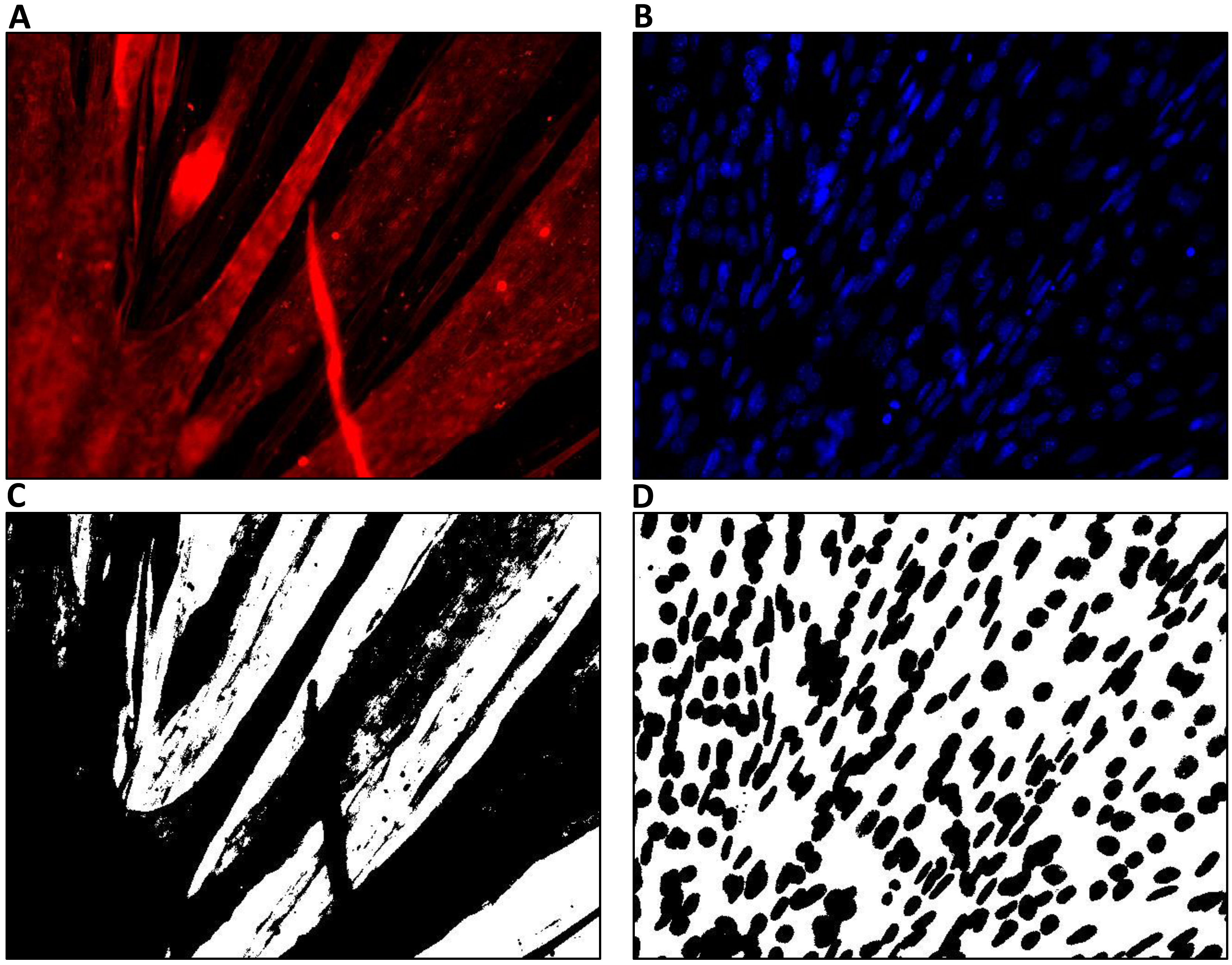

Supplement: Supplementary file 1 — Additional file 1: Figure S1. Representative masked images used for D6 myotube area calculation. (A) Myosin heavy chain, a marker of terminal differentiation, was visualized with the F59 antibody and an AlexaFluor568-linked secondary antibody. (B) DNA was visualized by DAPI staining. (C) The binary myosin mask corresponding to Panel A. (D) The binary DNA mask corresponding to Panel B. [file 13395_2019_211_MOESM1_ESM.tif]

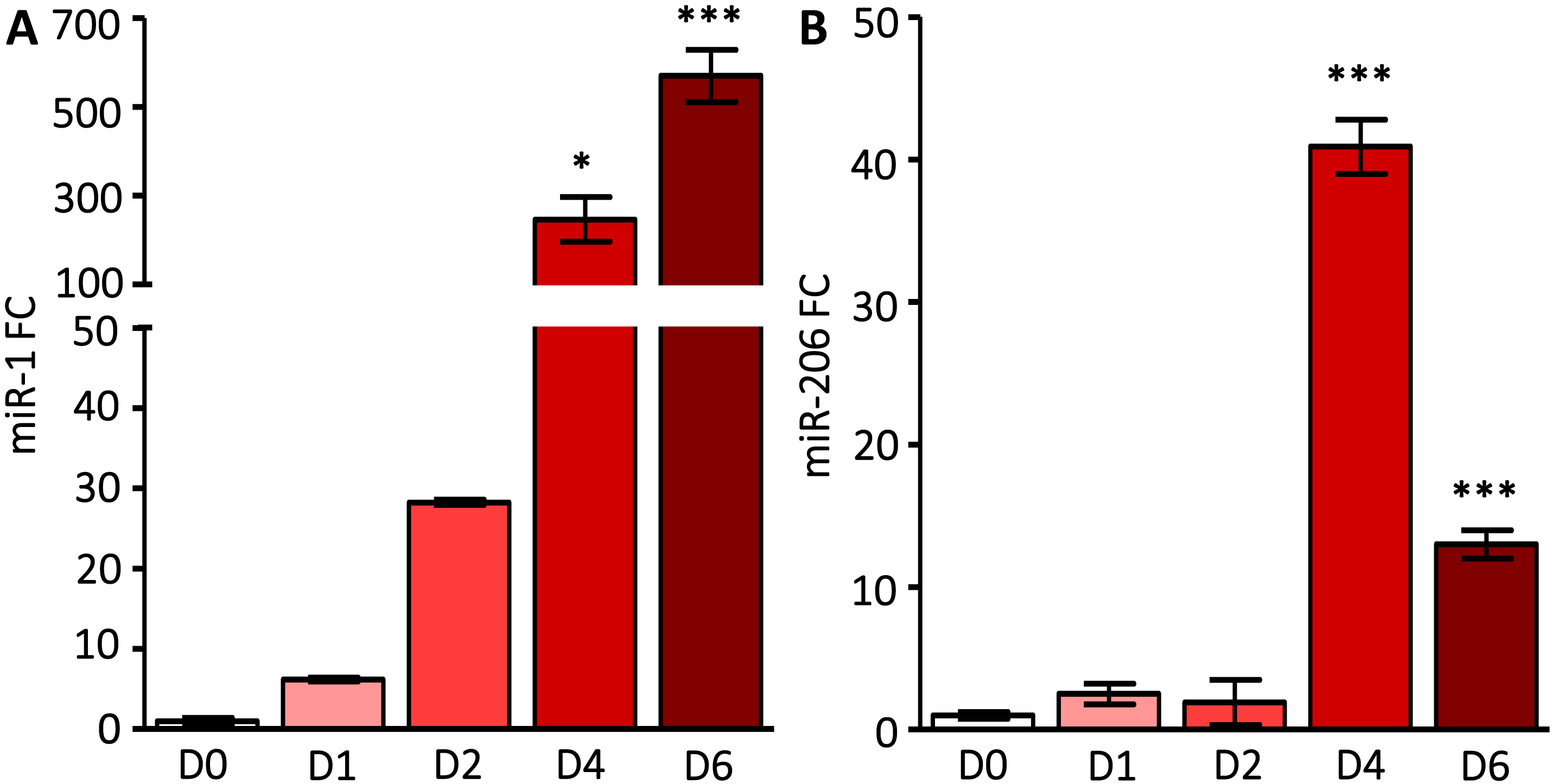

Supplement: Supplementary file 2 — Additional file 2: Figure S2. miR-1 and miR-206 levels increase during C2C12 differentiation. Expression was assessed by qPCR and normalized to sno202 in growing myoblasts (D0) and days 1, 2, 4, and 6 of differentiation. Fold changes vs D0 are presented. * = p < 0.05; *** = p < 0.001 [file 13395_2019_211_MOESM2_ESM.tif]

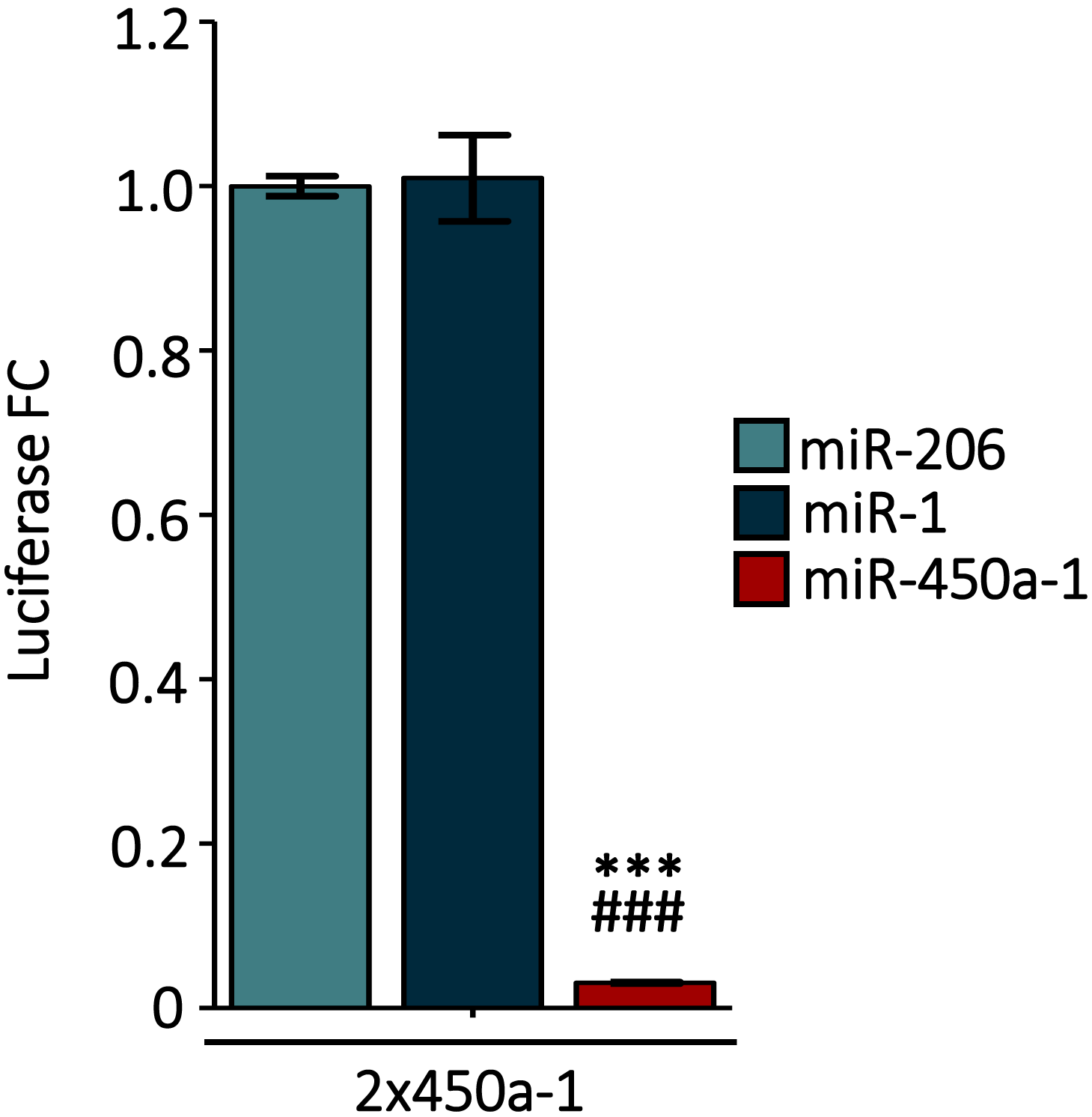

Supplement: Supplementary file 3 — Additional file 3: Figure S3. Non-myogenic miR-450a-1 is processed and active when ectopically expressed in myoblasts. C2C12 myoblasts were co-transfected with a reporter construct containing two tandem copies of the reverse complement of miR-450a-1 (2x450a-1) in the 3’ UTR along with expression constructs for miR-206, miR-1, or miR-450a-1. Cells were harvested 24 hours later. Firefly-normalized Renilla luciferase activity was equivalent between miR-206 and miR-1 expression but significantly down-regulated in the presence of miR-450a-1. *** = p < 0.001 vs miR-206; ### = p < 0.001 vs miR-1 N = 3 independent cultures for each. [file 13395_2019_211_MOESM3_ESM.tif]

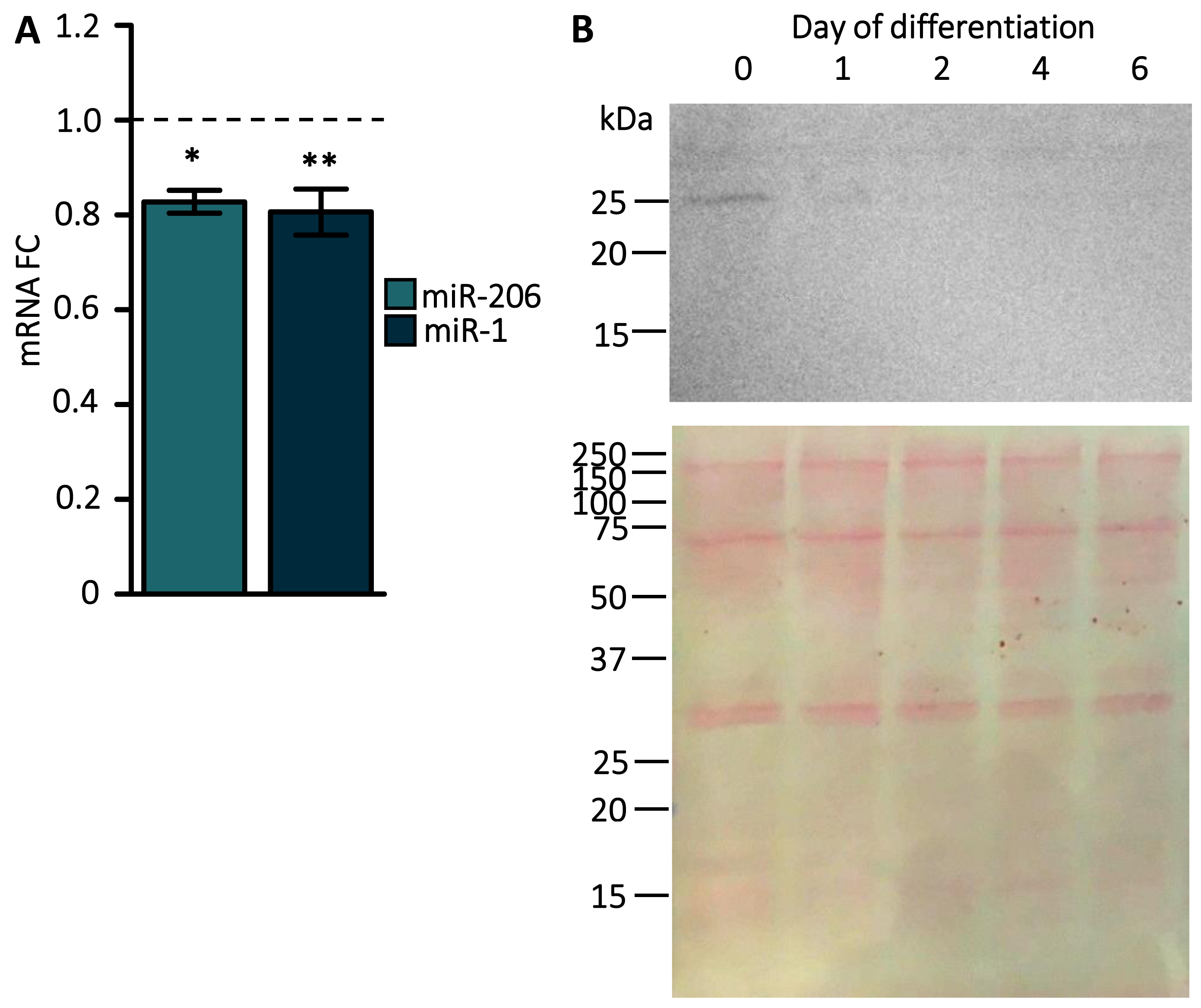

Supplement: Supplementary file 4 — Additional file 4: Figure S4. Endogenous Srsf9 mRNA and protein decrease with increased miR-1/206 expression. (A) Srsf9 mRNA levels decrease in C2C12 myoblasts transfected with miR-1 or miR-206 overexpression plasmids. Expression was assessed by qPCR and 18S rRNA was the reference gene. Expression levels were normalized to control myoblast levels (indicated by the dashed line). * = p ≤ 0.05; ** = p ≤ 0.01 vs. Control myoblasts. N = 3 independent cultures for each condition. (B) Srsf9 protein levels decrease in differentiating C2C12. Srsf9 was detected by western blot with a Srsf9-specific antibody (top panel). Ponceau S staining indicates equal protein loading (bottom panel). [file 13395_2019_211_MOESM4_ESM.tif]

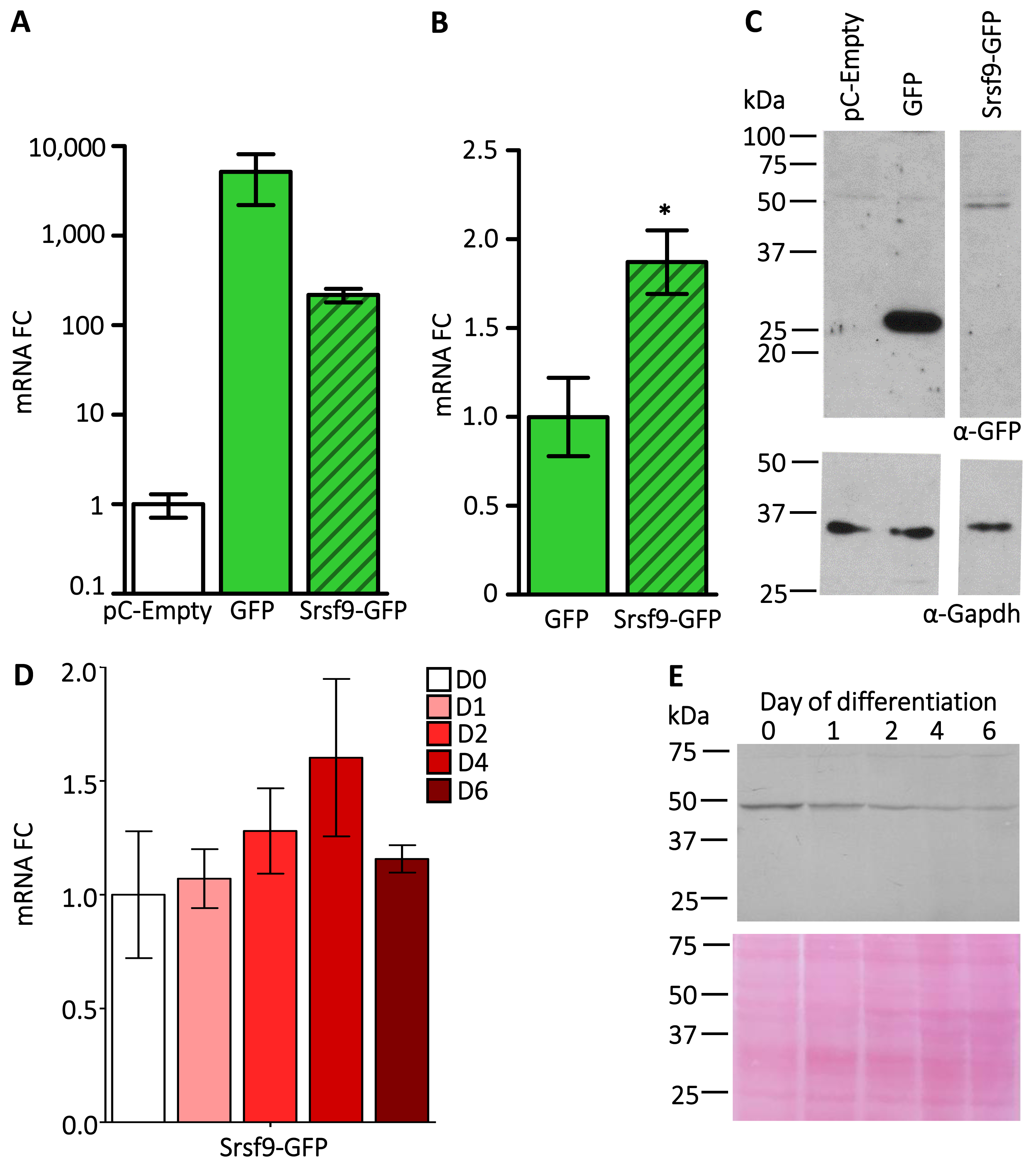

Supplement: Supplementary file 5 — Additional file 5: Figure S5. miR-1/206-resistant Srsf9-GFP expression does not change during differentiation of stable cell lines. (A) GFP or Srsf9-GFP mRNA levels in the corresponding stable C2C12 cell lines were compared to a negative control C2C12 cell line (pC-Empty) incorporating the empty expression vector pCDNA3.1(-). Expression was assessed by qPCR with GFP-specific primers and normalized to 18S rRNA levels. Fold change relative to pC-Empty is presented. N = 3 independent cultures for each. (B) Total Srsf9 (endogenous plus Srsf9-GFP) in Srsf9-GFP myoblasts is only 1.7-fold higher than endogenous Srsf9 levels in GFP control myoblasts. Expression was assessed by qPCR with Srsf9-specific primers and normalized to 18S rRNA levels. Fold change relative to GFP Control is presented. N = 3 independent cultures for each. * = p < 0.05 (C) Srsf9-GFP protein is expressed in the stable cell line. Expression in pC-Empty, GFP, and Srsf9-GFP cells was assessed by western blot with a GFP-specific antibody. (D) Srsf9-GFP mRNA levels are stable during differentiation of the Srsf9-GFP cell line. Expression was assessed by qPCR as in A. There is no statistical difference amongst the time points. (E) Srsf9-GFP protein levels are stable during differentiation of the Srsf9-GFP cell line. Expression was assessed by in-gel GFP autofluorescence (top panel). The gel was subsequently transferred to nitrocellulose which was then Ponceau S stained to reveal equal total protein loading (bottom panel). [file 13395_2019_211_MOESM5_ESM.tif]
